# Developing a protocol on antimicrobial resistance through WHO’s pandemic treaty will protect lives in future pandemics

**DOI:** 10.1186/s12992-024-01015-1

**Published:** 2024-01-31

**Authors:** Arne Ruckert, Shajoe Lake, Susan Rogers Van Katwyk

**Affiliations:** Global Strategy Lab, School of Global Health, York University, M3J 1P3 Toronto, ON Canada

## Abstract

Addressing antimicrobial resistance (AMR) through the pandemic treaty is a crucial aspect of pandemic prevention, preparedness, and response. At the moment, AMR-related provisions in the draft text do not go far enough and will likely lead countries to commit to the status-quo of AMR action. We suggest that the protocol mechanism of the treaty proposed under Article 31 offers an opportunity to develop a subsidiary agreement (or protocol) to further codify the specific obligations and enforcement mechanisms necessary to meet the treaty’s AMR provisions. We also highlight experiences with previous treaty implementation that relied on protocols to inform design of a future AMR protocol.

## Introduction

Antimicrobial resistance (AMR) is a natural evolutionary process, but can be accelerated by human activity, and occurs when bacteria, viruses, fungi and parasites no longer respond to antimicrobial medicines. AMR represents a key global governance challenge that requires equitable global coordination [1]. Existing governance mechanisms, including the International Health Regulations (IHRs) are limited in their ability to address AMR amidst deep fragmentation, insufficient governance infrastructure, and concerning global health inequities [2, 3]. Addressing AMR through the pandemic treaty must be a crucial aspect of pandemic prevention, preparedness, and response [4]. With the World Health Assembly’s May 2024 deadline for a pandemic treaty fast approaching, the Intergovernmental Negotiating Body recently released the fourth draft of the text [5]. While AMR is mentioned twice, the current text is not sufficient to safeguard the effectiveness of antimicrobials. The new pandemic treaty offers a path forward: the proposed protocol mechanism under consideration as Article 31 creates an opportunity to mitigate the impact of AMR on pandemic prevention, preparedness, and response [6, 7].^1^

Antimicrobials are a vital resource that must be preserved for responding to pandemic emergencies, as well as a potential source of future pandemics. At the same time, the use of antimicrobials during such emergencies may worsen AMR, with bacterial AMR estimated to globally have caused 1.29 million deaths, and being associated with almost five million deaths, in 2019 [8]. The latest draft of the pandemic treaty requires countries under Article 4(4)(g) “to take actions to prevent outbreaks due to pathogens that are resistant to antimicrobial agents, and, in accordance with national context, develop and implement a national One Health [OH] action plan that includes an antimicrobial resistance component” [5]. These provisions provide a starting point but are too general to lead to the effective implementation of the necessary policy actions, and will likely lead countries to recommit to the status quo of AMR actions– including limited implementation and financing of AMR national action plans by states, and unclear AMR obligations for non-state actors in terms of managing antimicrobial use. Global policy coordination, to date, has been insufficient to address a One Health challenge of this magnitude. Creating a pandemic treaty without adequately addressing AMR would be counter-productive as life-saving antimicrobials can help manage the burden of future pandemic threats, including through the treatment of secondary bacterial infections often associated with pandemics [9, 10]. The protocol mechanism offers an opportunity to develop a subsidiary agreement to codify the specific obligations and enforcement mechanisms [11] necessary to meet the treaty’s AMR provisions [12].

## An AMR protocol must address three key policy challenges

Protocols are subsidiary formal agreements that often supplement, clarify, or provide additional provisions for general obligations outlined in the main treaty. While protocols operate as separate legal instruments, they are designed to be integrated with, and interpreted in conjunction with the main treaty text. An AMR protocol could be negotiated and adopted simultaneously, or subsequently to the pandemic treaty, and designed to address three of the most complex AMR policy challenges that require sustained global collaboration: the procedures and mechanisms to address antimicrobial stewardship; facilitating effective One Health surveillance systems; and building capacity for treaty implementation. Many existing treaties have used the protocol mechanisms to provide more detailed guidance for the implementation of treaty provisions, by outlining clear obligations and enforcement mechanisms [11]. As described below, design features of an AMR protocol under the pandemic treaty could be informed by the experiences of successful protocol use to advance treaty goals in the areas of stewardship, surveillance, and capacity building.

### Stewardship of antimicrobials

Safeguarding the effectiveness of antimicrobials is essential to support global policy responses to future pandemics. An AMR protocol could develop globally harmonized rules governing which antimicrobials should be accessed, monitored, and reserved in national health systems. This would include developing a framework governing the stewardship of antimicrobials, to regulate the sustainable, acceptable, fair, and effective use of antimicrobials in health care, and limit the agricultural use of antimicrobials that are critically important for human health [13]. This would allow countries to explicitly adopt the World Health Organization’s (WHO) AWaRe framework which is the WHO classification system for antibiotics that guides the accessibility, monitoring, and reservation of antimicrobials [14].

There are experiences with previous protocols developed to achieve specific treaty goals related to stewardship that could inform design of an AMR protocol. For example, the *Agreement on the Conservation and Sustainable Use of Marine Biological Diversity of Areas Beyond National Jurisdiction* (“the BBNJ Agreement”) to the United Nations Convention on the Law of Sea (UNCLOS) created a procedure to conserve and manage the sustainable use of marine genetic resources in accordance with the UNCLOS conservation mandate [15]. Lessons from this process could inform formulation of specific obligations related to stewardship and sustainable use of antimicrobials, especially in terms of identification of, and agreement over, areas relevant for antibiotic stewardship and development of AMR management plans.

### One Health surveillance

Globally coordinated surveillance at the human/animal/plant/environment interface and beyond is essential for identifying novel pandemic threats, and the protocol must develop mechanisms for aligning surveillance and monitoring of emerging resistant bacteria with surveillance for other pathogens. Relying on the International Health Regulation’s Public Health Emergency of International Concern (PHEIC) system for notification of new resistant bacteria, as the current treaty text suggests, could overwhelm the IHR system with the sheer number of reports of new resistant outbreaks, while few of these individual outbreaks would meet the criteria for a PHEIC [3]. A protocol mechanism could establish minimum core One Health capacities for surveillance and monitoring and provide an additional framework for reporting of emerging strains of resistant bacteria [16, 17], which could further support the research and development of new antimicrobials [18].

In the area of surveillance, The *Protocol on Water and Health to the Convention on the Protection and Use of Transboundary Watercourses and International Lakes* represents a successful attempt to address a resource problem by establishing a regional framework for waste-water surveillance and environmental management, yielding insights into how to effectively align policies and strategies across various sectors for the protection of health, education, development, and the environment [19]. The process by which the protocol was developed could function as a template where as a first step a protocol outlines a surveillance framework and policy guidance, with the specific details of monitoring and surveillance programs left to the discretion of the individual Parties to the treaty. This approach would foster a step-wise collaborative approach to monitoring and surveillance, allowing countries to share information and best practices to address common challenges related to AMR.

### Capacity building

An AMR protocol should establish mechanisms for providing technical assistance and capacity-building to support countries that lack the technical capacity or resources to comply with the main treaty. This would make an important contribution to addressing capacity constraints for treaty implementations linked to inequitable resource endowments. Countries could agree upon sustainable financing mechanisms that specifically support increased development of laboratory infrastructure in low- and middle-income countries (LMICs) and facilitate knowledge and information sharing between LMICs and high-income countries [20]. These core capacities are critical for addressing AMR, especially to scale up surveillance in LMICs. While some measure of capacity development might be addressed in the pandemic treaty, the protocol could plug AMR-specific gaps.

In the area of capacity building, the *Montreal Protocol on Substances that Deplete the Ozone Layer* is a good example for how a protocol can facilitate treaty participation in resource challenged contexts. In addition to outlining specific obligations regarding the phasing down of ozone depleting substances in alignment with the United Nations Environment Programme, the *Montreal Protocol* mandated the creation of a fund and stipulated its purpose, beneficiaries, and contributors. Specifically, this fund was established to support LMIC implementation of the Protocol [21]. This multilateral fund has been shown to be an important incentive for LMICs to comply with treaty obligations, contributing to the protocol’s success, by promoting phase-out management plans (PMPs) for use of ozone-depleting substances via financial incentives [22]. Similar incentives could be used for promoting the phase out of antimicrobials in LMICs. Such a fund would also make an important contribution to address equity considerations in AMR governance as it would embody a resource transfer from the global North to the global South.

## Conclusion

By addressing these three key policy challenges through global collaboration, an AMR protocol can contribute to pandemic prevention and preparedness. A robust and globally coordinated surveillance system can facilitate early detection of trends in resistance to identify areas for proactive measures to address resistance before it becomes widespread, and thus contribute to pandemic prevention efforts. Developing a globally coordinated antimicrobial stewardship framework further contributes to pandemic preparedness by safeguarding the effectiveness of antimicrobial treatments, ensuring they are available as a treatment option in the face of future pandemics. Capacity building efforts will ensure implementation of surveillance and stewardship policies in resource constrained settings where the next pandemic is likely to hit the hardest.

Safeguarding antimicrobials and ensuring that they are equitably available to all is an essential aspect of comprehensive pandemic prevention, preparedness, and response. The protocol mechanism presents an opportunity to unify the global response by setting up structures that support sustained global collaboration and leverage technical expertise to ensure that interventions are evidence-based and protect lives in the face of future pandemics. However, in our global policy response to AMR, we need to go beyond the technical details and guidelines that would be expected from an AMR protocol, as there are significant macro-economic and socio-structural challenges that will require consideration of power differentials and inequities to design effective AMR policies at a global scale.

## Data Availability

There are no data or additional materials available related to this commentary.

## Footnotes

1 The relevance of the pandemic treaty to addressing AMR has previously been established [1, 4]. We are assuming for the purpose of this commentary that the pandemic treaty will impact global health governance practices, by facilitating better global collaboration and coordination in policy areas relevant to pandemic prevention, preparedness, and response.
